# High-Throughput Calculation of Interlayer van der Waals Forces Validated with Experimental Measurements

**DOI:** 10.34133/2022/9765121

**Published:** 2022-03-22

**Authors:** Kewei Tang, Weihong Qi, Yaru Wei, Guoliang Ru, Weimin Liu

**Affiliations:** ^1^State Key Laboratory of Solidification Processing, Center of Advanced Lubrication and Seal Materials, Northwestern Polytechnical University, Xi'an 710072, China; ^2^State Key Laboratory of Solid Lubrication, Lanzhou Institute of Chemical Physics, Chinese Academy of Sciences, Lanzhou 730000, China

## Abstract

Interlayer van der Waals interactions play an important role in two-dimensional (2D) materials on various occasions. The interlayer binding force is often directly measured and is considered more closely related to the exfoliation condition. However, a binding force database from accurate theoretical calculations does not yet exist. In this work, the critical interlayer binding force and energy are directly calculated for 230 2D materials, which exhibit divergent trends. A linear relationship that links the two quantities with the equilibrium interlayer distance is found and checked. Experiments are carried out for three different materials using atomic force microscopy. The measured forces show a consistent trend with the calculated results, and the estimated binding strengths are of the same order of magnitude as the predicted values. Our work can provide a reliable reference for interlayer adhesion studies and help establish accurate models of exfoliation processes.

## 1. Introduction

Interest in two-dimensional (2D) materials continues to grow due to their promising properties and the ability to arbitrarily integrate themselves [1, 2]. The latter takes advantage of comparatively weak interlayer van der Waals (vdW) interactions. The interlayer vdW interaction is far weaker than ionic bonding [3] but strong enough to hold layers together and enable a certain degree of electronic coupling at the interface. This characteristic of interlayer vdW interactions has enabled many fascinating properties and novel physical phenomena, such as flat-band and van-Hove singularities [4–6]. From perturbation theory, the vdW potential is found to be a series expansion: −∑_*n*≥3_^∞^*f*_2*n*_(**R**)*C*_2*n*_^2^**R**^−2*n*^ (where **R** is the distance) that should be terminated at different terms for different materials to achieve a certain accuracy [7, 8]. While taking only the first term of the series expansion as the vdW potential and modeling the exchange potential with an inverse power term, one obtains the famous Lennard–Jones (L-J) potential. Similarly, if the exchange potential is modeled more accurately with the Born–Mayer term [9], then the well-known Buckingham potential is obtained. Moreover, there are still many other potential models extended from these two models [10–13]. These models are very simple in form. However, in complex systems, the vdW interaction can be affected by the local bonding environment and screening effect [14, 15], thus making it harder to correctly characterize it with semiempirical methods. In general, the most acknowledged method to characterize accurate vdW interactions is still perturbation theory (PT) calculations [16, 17].

One of the basic concerns related to the interlayer vdW interaction is exfoliation. Examining all the available exfoliation technologies, one would find that exfoliation is always a complicated process in the sense of mechanics [18]. The detailed mechanical process is very important but rarely visited. To obtain a general sense of the difficulty level of exfoliation, exfoliation energy is widely used, which is defined as the energy to extract one layer from a bulk material [19]. In calculations or simulations, interlayer binding energies are used instead since they are more computationally convenient (the two concepts are closely related and differ slightly in value [17]). While the binding energy carries the information for how much energy is needed to complete exfoliation when exfoliation is known to succeed, it cannot directly give the critical mechanical condition. However, while the interlayer binding energy (or cohesive energy in some cases) was used, calculated, and measured in many cases, the interlayer binding force usually only receives its attention in fundamental studies focusing on interlayer interaction [14, 20].

For 2D materials, although adhesion energy measurement is viable with fracture mechanics approaches [21], adhesion force measurement is often more direct and intuitive. As a result, many works study interlayer binding by measuring the force and converting it to energy with approximate models. For example, a recent study measured the critical force to split a square mesa made of 2D materials using atomic force microscopy (AFM), and the interfacial adhesion energy was obtained by integrating the retraction curves [22]. This can be seen as an approximation to the integration of the real interlayer force curve, but it can be significantly overestimated (especially when the spring constant of the probe is low). More reliable measurements often rely on classical continuum mechanics models such as JKR (Johnson-Kendall-Roberts) [23], DMT (Derjaguin-Muller-Toporov) [24], Maugis-Dugdale [25], and MYD (Muller-Yushchenko-Derjaguin) [26] to extract adhesion energy from pull-off force [27–29]. The JKR model includes only the “inside adhesion” (within a defined area) and is only applicable for soft substrates. The DMT model uses a Hertzian profile for repulsion, which is far from realistic and is only applicable for very hard substrates. The Maugis-Dugdale model uses a simple Dugdale model and has a variable coefficient that can be tuned for different circumstances. The Maugis-Dugdale model can be reduced to either the JKR or the DMT models by tuning this coefficient and can be viewed as a transitional model between these two models. Obviously, the determination of the coefficient is vital to the accuracy of the model. Since it cannot be derived solely from force-distance curves, many studies obtain this coefficient by fitting an empirical equation proposed by Carpick et al. [30]. The MYD model is another step taken for filling the gaps between the JKR and DMT models by using the Lennard–Jones potential, which is considered more realistic. Similarly, there are parameters that must be predetermined for MYD-like models, such as the equilibrium distance. Different models can be chosen for different cases to reach optimal accuracy. However, these models are approximations and can sometimes induce unexpected errors. Adhesion energies measured using these models are often not comparable to other similar studies due to the differences in experimental details (such as tip curvature), and the directly measured forces are not compared to reliable theoretical references.

Reliable and abundant interlayer force data from accurate theoretical predictions are urgently needed. In this study, we aim to provide such a reliable data set with reliable first-principle calculations. Targeting 230 different common 2D materials provided by Mounet's 2D material database [19], a simple effective descent algorithm is designed and implemented to work with the Vienna Ab initio Simulation Package (VASP) code to perform first-principle calculations for the critical interlayer binding force *F*_*b*_ and energy *E*_*b*_. Both the optB88-vdW and vdW-DF2 functionals are used, and the results are compared with accurate random phase approximation (RPA) calculations. Experimental measurements are carried out by coating 2D materials onto homemade silica probes and measuring the force between homogeneous 2D material interfaces in AFM. The predicted results show significant divergence between the trend of binding force and binding energy, which emphasizes the irreducibility between the two quantities and hence the meaning for providing the binding force data. The measured data show excellent agreement with the predictions.

## 2. Results

### 2.1. Prediction of Interlayer Binding Energy and Force

**Figure 1:**
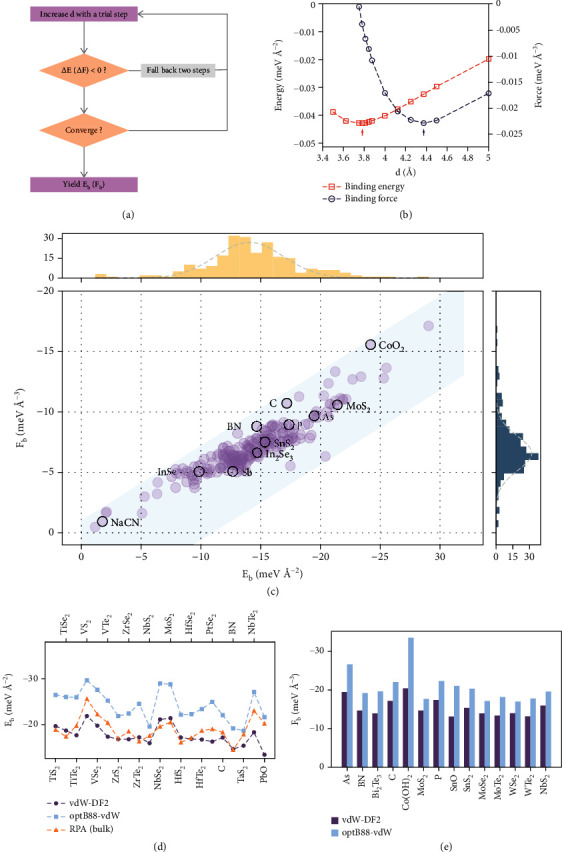
Calculation of bilayer *F*_*b*_ and *E*_*b*_. (a) Flow chart of a simple descent algorithm to locate *E*_*b*_ and *F*_*b*_ (d is the interlayer distance). (b) Example outputs (for 2H-MoS_2_) of the steps generated by the algorithm. (c) *E*_*b*_ and *F*_*b*_ calculated for 230 different 2D materials with the vdW-DF2 functional. (d) Comparison of the calculated bilayer *E*_*b*_ with the vdW-DF2 and optB88-vdW functionals and the bulk *E*_*b*_ calculated with RPA [17]. (e) Comparison of the forces calculated with both functionals for a few common 2D materials.

To locate the minimum interlayer binding force and energy, we present a simple descent algorithm, as shown briefly in Figure 1(a). The binding energy and force are calculated in bilayers, which is different from many other works since the binding force in the bulk structure cannot be calculated with periodic boundary conditions. To capture the dispersive interaction [17], two well-known nonlocal vdW functionals are adopted: optB88 (an optimized vdW-DF functional) [31] and the vdW-DF2 [32] functional. An example output with the vdW-DF2 functional of the algorithm implementation is shown in Figure 1(b).

Figure 1(c) shows the *E*_*b*_ and *F*_*b*_ calculated with the vdW-DF2 functional for 230 different 2D materials provided by Mounet's 2D database [19], which is marked as “easily exfoliable.” The data points are located within a band around a straight line, which indicates loose linear-like dependencies between *F*_*b*_ and *E*_*b*_ with notable divergence. The calculated *E*_*b*_ and *F*_*b*_ for many materials share a similar trend because they are gathered in the center of the band where the data points severely overlap with each other. However, significant divergence can be found for many systems located away from the center. For example, two different 2D materials with the same or similar binding energies can possess a difference in binding forces as large as 5 eV Å^−3^. For quite a few of the systems, the trend of *E*_*b*_ can even go against the trend of *F*_*b*_ (C and As e.g.). The results calculated with the optB88-vdW functional show a similar divergence (which can be found in Supplementary Figure 1). The subplots at the top and right sides of Figure 1(c) show the distribution of the two quantities. More than 80% of the calculated 2D materials possess interlayer binding energy within the range of -10 to -20 eV Å^−2^; approximately 90% of the calculated 2D materials possess interlayer binding force within the range of -5 to -10 eV Å^−3^. *E*_*b*_ with the equilibrium distance *d*_0_ and *F*_*b*_ with the critical distance *d*_1_ calculated from the vdW-DF2 functional for a few common 2D materials are listed in Table 1, and the full results calculated with the vdW-DF2 and optB88-vdW functionals can be found in Supplementary Table 1 and Supplementary Table 2, respectively. The distribution of *d*_0_ and *d*_1_ is shown in Supplementary Figure 4.

It is worth noting from the calculated results that the equilibrium interlayer distance calculated with vdW-DF2 might be slightly overestimated, as it has been reported as one of the drawbacks of the vdW-DF methods [17, 33, 34]. Taking graphene as an example, the experimentally observed interlayer distance for graphite is approximately 3.35 Å, which is significantly smaller than the predicted value of 3.66 Å. However, one should be aware that the experimental value is given for the A-B stacking mode, while the predicted value is for the A-A stacking mode, and the A-A stacking mode usually results in a discernably larger distance due to stronger repulsion. This difference caused by the stacking mode has been demonstrated by other studies [33, 34]. A reliable reference for the A-A stacking interlayer distance is 3.42 Å from the RPA calculation for bulk graphite [33]. Considering that the interlayer distance increases with the decrease in the number of layers [35], the actual overestimation of the predicted value in this study should be notably less than 7.0%. These assessments suggest that the distances calculated can be slightly overestimated, but not significantly.

The *E*_*b*_ calculated from the two different vdW functionals are compared with each other and with the results calculated from an RPA calculation [17] for some systems. The comparison is shown in Figure 1(d). Obviously, both results share a similar trend with that of the RPA calculations. However, the *E*_*b*_ calculated with the vdW-DF2 functional is closer to the RPA results, while the *E*_*b*_ calculated with the optB88-vdW functional is notably larger than that of the vdW-DF2 functional. This drawback of the optB88-vdW functional has also been reported by other studies [36, 37]. The vdW-DF2 results are slightly lower than the RPA references since the RPA results are for bulk systems. Figure 1(e) shows the comparison between the forces calculated with the two functionals. It is obvious that optB88-vdW tends to overestimate *F*_*b*_ as well, but the overestimation is slighter.

### 2.2. The Loose Relationship between *E*_*b*_, *F*_*b*_, and *d*_0_

**Figure 2:**
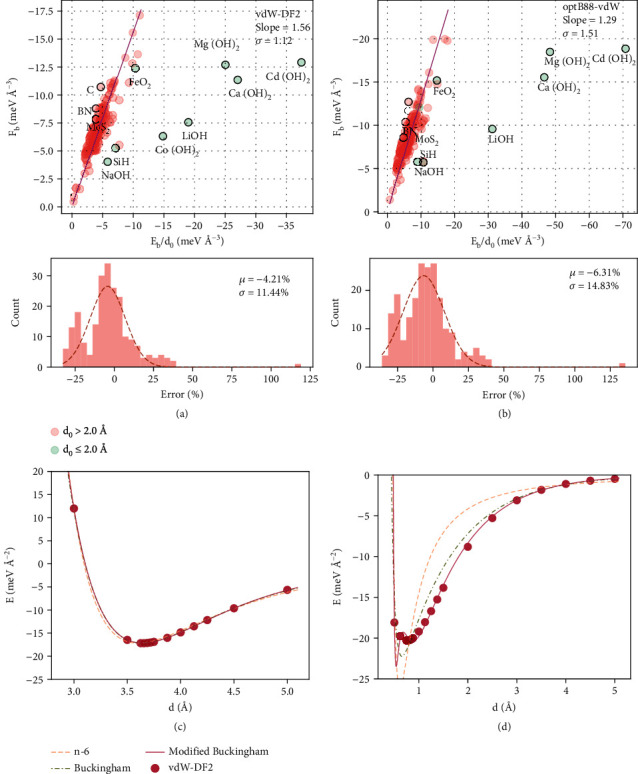
Fitting the loose relationship derived from the n-6 potential. (a) Fitting with the vdW-DF2 results. (b) Fitting with the optB88-vdW results. The fitted slope and the standard deviation are shown in the top right corners of the main plots, and the force error distribution (*η*(*E*_*b*_/*d*_0_) − *F*_*b*_) is shown below the main plots. (c) Fitting three different potential models with the binding energies of graphene. (d) Fitting three different potential models with the binding energies of Ca(OH)_2_. (*μ* and *σ* represent the expected value and standard deviation, respectively).

Assuming the interlayer binding energy is modeled by the traditional n-6 potential where the repulsion term takes the form of *Ar*^−*n*^, the interlayer binding force *F*(*d*) is then represented as its derivative. By solving *F*(*d*) = 0, a simple relationship between *E*_*b*_ and *F*_*b*_ is then achieved (details can be found in the Supplementary Information),

(1) $$\begin{array}{c} F_{b}=\eta \cdot \frac{E_{b}}{d_{0}}, \end{array}$$

where *η* is a constant determined by *n*. Although the n-6 potential might not fit equally well for all kinds of 2D materials, this expression might still be good for rough estimations. To verify this idea, the calculated *F*_*b*_ and *E*_*b*_/*d*_0_ are fitted with a line through the origin, as shown in Figure 2(a) and Figure 2(b) for the vdW-DF2 and optB88-vdW results, respectively. During the fitting of the data, it was found that the data points with small *d*_0_ tend to stray away from the main trend, while other data points fit comparatively well with the line. These abnormal data points with exceptionally small *d*_0_ values, as labeled in the graph, are mostly 2D hydroxides, which in their natural state should have hydrates in between layers. It should be noted that the results presented here do not involve hydrates between layers. To obtain a clearer trend of the data, only the data points with *d*_0_ larger than 2 Å are included in the fitting. For the vdW-DF2 results, most of the data points are gathered closely (with the standard deviation being just 1.12 eV Å^−3^) around the fitting line with a slope of 1.56. Similar trends can be seen for the optB88-vdW results, but the slope is much smaller (1.29), which can be attributed to the significantly overestimated *E*_*b*_ compared to the slight overestimation of *F*_*b*_, as mentioned above. The good agreement with the calculated results suggests that equation (1) can be used to roughly predict the interlayer binding force from the equilibrium distance and binding energy.

As the expression is derived solely from the n-6 potential, the deviation might infer the inherent inaccuracy of the potential model. To determine the details, the interlayer binding energies produced by the descent algorithm are fitted with various potential models. Here, we choose Ca(OH)_2_ ($d_{0}=0.75\;\overset{{}^{\circ}}{\text{A}}$, which cannot fit in Figure 1(a)) and graphene (which fits well in Figure 1(a)) from the vdW-DF2 calculations to fit three potential models: the n-6 model, the Buckingham model, and the modified Buckingham model [38]. The expressions of the three potential models are shown in the following equations:

(2) $$\begin{array}{c} E\left(d\right)=A\cdot \left[\frac{6}{C-6}{\left(\frac{B}{d}\right)}^{C}-\frac{C}{C-6}{\left(\frac{B}{d}\right)}^{6}\right], \end{array}$$

(3) $$\begin{array}{c} E\left(d\right)=A\cdot e^{-B\cdot d}-\frac{C}{d^{6}}, \end{array}$$

(4) $$\begin{array}{c} E\left(d\right)=A\cdot e^{-B\cdot d}-\frac{C_{1}}{d^{6}}-\frac{C_{2}}{d^{8}}-\frac{C_{3}}{d^{10}}, \end{array}$$

where *A*, *B*, *C*, *C*_1_, *C*_2,_ and *C*_3_ are constant parameters to be determined. Global optimization is used to search for the best parameters. The fitting results of C and Ca(OH)_2_ are shown in Figure 2(c) and Figure 2(d), respectively. The n-6 potential fits well for graphene but is comparatively poor for Ca(OH)_2_, which may be the reason why the Ca(OH)_2_ strays from the main trend in Figure 1(a). The failure of the n-6 potential in Ca(OH)_2_ is common in systems with small *d*_0_. In such cases, the variation in the exchange energy can be very drastic, whereas the Born–Mayer term has an exponential form, *A* · *e*^−*B*·*d*^ can model the short-range exchange effect more reasonably, thus fitting better for many systems [9, 39–41]. The Buckingham potential is just a direct modification from the n-6 potential by replacing the *A* · *e*^−*B*·*d*^ term with the Born–Mayer term. The fitting with the Buckingham potential in Figure 2(d) shows a notable improvement. Moreover, having more dispersive terms, such as the modified Buckingham potential in equation (4), can be advantageous, as shown in Figure 2(d), but the benefits are less significant. These analyses demonstrate that the deviation of the calculated results from the proposed relationship (equation (1)) is mainly due to the inherent inaccuracy brought by the inverse-power repelling term. For this reason, models based on n-6-like potentials (such as the L-J potential) for converting *E*_*b*_ to *F*_*b*_ or vice versa (the Maugis model [25], e.g.) should not be considered accurate methods. This applies to the loose relationship presented in equation (1), which should only be used for rough estimations.

### 2.3. Experimental Measurements of Interlayer Cohesive Force

**Figure 3:**
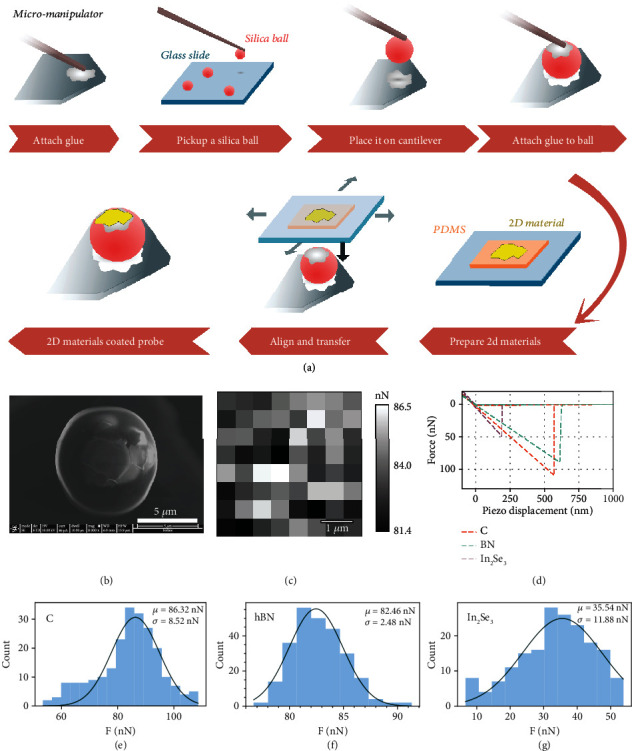
Experimental measurements of the interlayer cohesive force with AFM. (a) Schematics of the fabrication of 2D-material-coated AFM probes. (b) SEM image of the probe coated with BN. (c) A typical force map of BN produced in AFM force measurements. The measured area is 4 × 4 *μ*m^2^ (very large compared to other studies) sampled with an 8 × 8 grid. (d) Typical trace and retrace force curves of C, BN, and In_2_Se_3_. The jump-off distance is comparatively large because the cantilevers have very low spring constants. (e–g) The distribution of the measured forces with Gaussian fit for C, BN, and In_2_Se_3_, where *μ* is the expected value and *σ* is the standard deviation. (*μ* and *σ* represent the expected value and standard deviation, respectively).

Experimental evidence is needed to confirm the validity of the predictions. Since corresponding measurements are few and some might be inaccurate, an experiment is designed by using atomic force microscopy (AFM) with 2D-material-coated probes. Three different 2D materials with chemical formulas of C, BN, and In_2_Se_3_ are chosen for measurements. The key point of this method is to fabricate AFM probes with appropriate tip curvature (silica balls are used to achieve this purpose) and wrap the 2D materials onto them. The probe fabrication process using glue for welding resembles the method reported by Li et al. [42], but a high-strength type of glue and a homemade micromanipulator are used, which enable us to fabricate the probes more efficiently and precisely. The results are shown in Figure 3. The fabrication process is shown in Figure 3(a), which is further illustrated in the methods section. The advantage of this fabrication process is that it will not contaminate the cantilevers with extra glue since all glue is applied precisely to where it needs to be using the micromanipulator, and the upward coating of 2D materials with soft substrates can make them wrap more seamlessly onto the ball.

The design of the measurement and the analysis of feasibility can be found in Section 2 of the Supplementary Information. Figures 3(e)–3(g) show the distribution of the measured data. The data fit very well against the Gaussian distribution, with the expected values of *F*_*C*_ = 86.32 ± 8.52 nN, *F*_*BN*_ = 82.46 ± 2.48 nN, and *F*_*In*_2_*Se*_3__ = 35.54 ± 11.88 nN. These values are then corrected for radius differences and converted to strength (per-unit-area forces) with an effective contact area of ~89.00 nm^2^ (details in Section 2.4 of the Supplementary Information), which is shown as a comparison to the calculated results in Table 2. The statistical results show a trend: *F*_*G*_ > *F*_*BN*_ > *F*_*In*_2_*Se*_3__, which is consistent with the theoretical prediction, and the values are of the same magnitude as those of other similar studies [20]. The corrected forces are very close to the uncorrected values since the radii of the silica balls are rather uniform. The estimated strengths are of the same magnitude as the calculation results, proving that the measured results are quite reliable and can correctly reflect the trend of the actual interlayer binding force.

Since the relationship predicted between the binding energies of the three different materials, *E*_*C*_ > *E*_*BN*_≅*E*_*In*_2_*Se*_3__, is consistent with other theoretical studies [17, 19], together with the force relationship measured as *F*_*G*_ > *F*_*BN*_ > *F*_*In*_2_*Se*_3__, the inconsistency between *E*_*b*_ and *F*_*b*_ is also proven. The *E*_*b*_ of BN and In_2_Se_3_ are similar, but the *F*_*b*_ differs quite notably. This inconsistency between *E*_*b*_ and *F*_*b*_ emphasizes that attention should be given to binding forces.

## 3. Discussion

In this study, high-throughput calculations are carried out for 230 2D materials to determine their maximum binding energies and forces. Three materials are chosen to perform experimental interlayer cohesion force measurements. The calculation results show excellent agreement with both accurate RPA calculations and the measured results. The predicted critical binding forces for 230 different 2D materials validated with experimental measurements can serve as a reliable source of references for future studies related to interlayer adhesion. It can be especially helpful for indirect measurements of interlayer adhesion energy from adhesion force since the data provided by this work can be used to check with the directly measured forces before conversion to ensure the correctness of the direct data in the first place. The proposed method for high-throughput calculation and experimental measurement of interlayer binding forces can be easily extended to other 2D materials. In a more general sense, we hope the results of this work can help future studies to better understand the characteristics of vdW interactions in 2D materials and to build accurate mechanical models for exfoliation.

## 4. Materials and Methods

### 4.1. Calculation Parameters for VASP Calls

The first-principle calculations were performed with the VASP [43] code using the projector-augmented plane-wave (PAW) method. The PBE [44] functional was adopted to address the exchange interaction, while the optB88-vdW [31] and vdW-DF2 [32] functionals were adopted to address the correlation interaction. For all the calculations, the energy cutoff of the plane-wave basis was set to 550 eV, the first Brillouin zone was sampled with a 15 × 15 × 1 Monkhorst Pack grid, and the convergence criteria for self-consistent field loops were set to 10^−5^ eV. For monolayer relaxation, only the in-plane adjustments were allowed with an atomic force tolerance of -0.01 eV Å^−1^. For static calculations of monolayers and bilayers, the highest accuracy has been enabled to achieve more accurate atomic forces.

### 4.2. Descent Algorithm for Locating *F*_*b*_ and *E*_*b*_

The algorithm assumes that the interlayer energy or force profile is a single-valley curve and searches for the minimum by ensuring that every step reduces the energy or force. Upon convergence, the maximum binding energy *E*_*b*_ is yielded at the equilibrium distance *d*_0_, while the critical binding force *F*_*b*_ is yielded at the critical distance *d*_1_. The algorithm is implemented in Python to work with VASP. The results yielded for demonstration are shown in Figure 1(b). For most cases, the algorithm is capable of locating *E*_*b*_ and *F*_*b*_ with approximately 15 VASP calls, which is efficient enough for this work. The details of how the routine was called are explained in the next subsection.

### 4.3. High-Throughput Calculation

The whole calculation was automated with Python (3.6.3) scripting, and the descent algorithm was implemented with Python. Bilayer systems are constructed with a custom Python library PyCrystal, which provides various lattice operations. For each 2D system, monolayer relaxation and monolayer static calculations were carried out first. Then, the bilayer systems were built with relaxed structures, and the descent algorithm was started to locate the minimum binding energy and atomic force. During the minimum search, both the energy and force data were produced. *E*_*b*_ is searched first, the algorithm starts from $d=5\;\overset{{}^{\circ}}{\text{A}}$ and searches toward $d=0\;\overset{{}^{\circ}}{\text{A}}$, with a convergence criterion of 10^−4^ eV per atom. Then, the produced data are fed to the search for *F*_*b*_, which starts from the deepest valley of the provided data. The criterion for convergence of *F*_*b*_ was set to 10^−3^ eV Å^−1^ per atom. To avoid local convergence while there are multiple valleys in the *E*(*d*) or *F*(*d*) profiles (there are a few such cases in the optB88-vdW calculations), a check for global convergence is performed before real convergence. To ensure sufficient numerical accuracy, the convergence criterion was set to 10^−4^ eV per atom for the binding energy and 10^−3^ eV Å^−1^ per atom for the binding force.

### 4.4. Global Search for Fitting Parameters

The simulated annealing algorithm for potential fitting is implemented in C. Exponential temperature management is used with a base value of 0.999, the initial temperature is 300 K, and the critical temperature is 10^−5^ K. The algorithm searches 10000 times in every temperature step and breaks at the critical temperature for divergence. Convergence is reached when the optimized values remain the same for 100 runs. During the fitting of the potentials, both the global fitting technology and the traditional trust-region-based local search are used together to ensure the best fitting results.

### 4.5. Fabrication of 2D-Material-Coated Probes

Before starting, a homemade micromanipulator with a tip size of ~10 *μ*m was equipped onto the 2D material transfer system. Using the micromanipulator, a small amount of epoxy glue was precisely applied on the pointed end of a tipless cantilever. Then, the micromanipulator was replaced with a clean one to take a silica ball with a radius of ~4.5 *μ*m from the glass slide (this is possible because the adhesion between the silica balls and the glass slide is much weaker than the adhesion between the micromanipulator and the silica balls). Aligning the silica ball on the micromanipulator with the previously glued cantilever, the silica ball was carefully attached to the cantilever. After the glue was dried (>24 h), a small amount of glue was precisely applied onto the top of the previously glued silica ball. Then, the 2D material flake prepared on polydimethylsiloxane (PDMS) was loaded in whole in the transfer system and slowly pressed against the ball probe while keeping the alignment at the same time. It is pressed down to a certain depth after initial contact to ensure full contact between the 2D material flake and silica ball. In this way, extra glue will be squeezed out, and the residual glue between the interface will be very thin so that its influence on the curvature of the 2D material flake can be neglected. Finally, the glass slide carrying the PDMS was immediately lifted to detach the 2D flake and leave it on the silica ball. The 2D flakes can detach from the PDMS since the glue has a very high tensile strength (sticky), even when it has not yet dried.

### 4.6. Interlayer Cohesion Force Measurements

The 2D material transfer system is E1-T, manufactured by MetaTest Co. Highly oriented pyrolytic graphite (HOPG) is produced by the Institute of Metals, Chinese Academy of Sciences; highly oriented hexagonal BN is purchased from Onway Technology Co.; *α*-In_2_Se_3_ (2H phase) is produced by HQ Graphene Co. The tipless cantilever is TL_CONT, Nanosensors. The spring constant given by the manufacturer is 0.02-0.77 N/m. High-quality silica balls were acquired from Nano-Micro, Co. All three different materials were characterized by X-ray diffraction (XRD). The results are shown in Supplementary Figure 8. The 2D material flakes were prepared with mechanical exfoliation using Scotch tape. The size (slightly smaller than the size of the silica balls) and thickness (>20 nm as demanded) of the flakes were filtered by AFM morphology characterization to find the flakes appropriate for coating. Smooth large flakes are selected and transferred onto silicon substrates using the 2D material transfer system. The epoxy glue used was T200 manufactured by Ergo Co., Ltd. The fabricated probes were characterized with scanning electron microscopy (SEM), and more images can be found in Supplementary Figure 9 and Supplementary Figure 10. The measurements were carried out using Asylum Research MFP-3D Origin+ AFM in tapping mode. For each kind of 2D material, 2 to 3 large flakes that are the smoothest were chosen as the substrate. On these selected flakes, several different areas (each with a size of 4 × 4 *μ*m^2^) were sampled in the measurements. The sample points without a successful jump-off were not accounted for as valid data and were not included in the statistics. All measurements were carried out under ambient conditions. The temperature was 21-27°C, and the relative humidity was 20%-25%. The humidity is very low, and the capillary effects are assumed to be negligible, as found in many studies [45–47]. In addition, no long-tail characteristic caused by water bridges [48] is found in the force-distance curves, which supports this assumption. Force measurements and SEM characterizations were performed alternately. After the first measurement and SEM characterization, a second measurement (for verification only) and SEM characterization were carried out. The measurement results before and after the first SEM characterization are highly identical, which supports that charge pollution induced by the electron beam is negligible in our case [47]. The SEM characterizations before and after the measurements are shown in Supplementary Figure 11.

## Figures and Tables

**Table 1:** *d*_0_, *E*_*b*_, *d*_1_, and *F*_*b*_ calculated with the vdW-DF2 functional for 14 common 2D materials.

Materials	Space group	*d*_0_ (Å)	*E*_*b*_ (meV Å^−2^)	*d*_1_ (Å)	*F*_*b*_ (meV Å^−3^)
As	Pmna	3.656	-19.4827	4.375	-9.648
BN	$\text{P}\bar{6}\text{m}2$	3.750	-14.6693	4.250	-8.793
Bi_2_Te_3_	$\text{P}\bar{3}\text{m}1$	3.563	-13.9344	4.500	-5.981
C	P6/mmm	3.656	-17.1861	4.125	-10.72
Co(OH)_2_	C2/m	1.375	-20.4132	3.000	-6.32
MoS_2_	$\text{P}\bar{6}\text{m}2$	3.781	-14.6763	4.375	-7.827
P	Pmna	3.750	-17.3697	4.500	-8.919
SnO	C2/m	3.875	-13.1113	4.500	-5.426
SnS_2_	$\text{P}\bar{3}\text{m}1$	3.125	-15.3632	4.000	-7.508
MoSe_2_	$\text{P}\bar{6}\text{m}2$	3.969	-13.9459	4.500	-7.035
MoTe_2_	P2_1_/m	3.719	-13.3608	4.500	-5.755
WSe_2_	$\text{P}\bar{6}\text{m}2$	3.938	-13.9994	4.500	-7.082
WTe_2_	P2_1_/m	3.719	-13.1701	4.500	-5.653
NbS_2_	$\text{P}\bar{6}\text{m}2$	3.563	-15.9550	4.125	-8.803

**Table 2:** The measurement forces compared with the predicted data.

Material	*R* (*μ*m)	Measured force (nN)	Corrected force∗ (nN)	Estimated strength (meV/Å^3^)	vdW-DF2 results (meV/Å^3^)	optB88-vdW results (meV/Å^3^)
C	4.44	86.32 ± 8.52	86.79 ± 8.56	6.02	10.72	12.70
BN	4.60	82.46 ± 2.48	80.11 ± 2.40	5.78	8.79	10.34
In_2_Se_3_	4.69	35.54 ± 11.88	34.52 ± 11.54	2.49	6.63	8.24

^∗^This is only the result of a rough correction based on the L-J model.

## Data Availability

The main data not shown in the main text can be found in the Supplementary Information. Other data, including the initial structures of the 2D materials, the data produced by each step of the algorithm, and the measurement results, were published in the Materials Cloud Archive (doi:10.24435/materialscloud:t0-hn). The implementation of the descent algorithm is a single file named ‘descender.py' in the data published in the Materials Cloud Archive. The code that automated the calculation process is not publicly available. The custom code PyCrystal is licensed under GPL v3.0, which can be found at https://github.com/CementMixer/pycrystal.

## Supplementary Materials

Supplementary Materials Supplementary Figure 1: *F*_*b*_-*E*_*b*_ calculated with the optB88-vdW functional. Supplementary Figure 2: distribution of energy error (*F*_*b*_*d*_0_/*η* − *E*_*b*_). Supplementary Figure 3: distribution of *F*_*b*_*d*_0_/*E*_*b*_. Supplementary Figure 4: distribution of the calculated *d*_0_ and *d*_1_. Supplementary Figure 5: diagram of the geometry for the integration model. Supplementary Figure 6: visualization of Supplementary equation (13). Supplementary Figure 7: visualization of *I*_1_ − *I*_2_ with *t* = 15 nm. Supplementary Figure 8: XRD characterization of the three different 2D materials. Supplementary Figure 9: SEM characterization (with BSE) of the three fabricated probes. Supplementary Figure 10: high-resolution SEM characterization (with SE) of the fabricated probes. Supplementary Figure 11: comparison of the coated 2D material flakes before and after measurements. Supplementary Table 1: the binding energy and binding force calculated with the vdW-DF2 functional. Supplementary Table 2: the binding energy and binding force calculated with the optB88-vdW functional.
