# Supplementary material for: High-Throughput Calculation of Interlayer van der Waals Forces Validated with Experimental Measurements
Source: Research (Wash D C). 2022 Mar 22;2022:9765121. doi: 10.34133/2022/9765121 (PMC8968625; doi:10.34133/2022/9765121)
Supplement: Supplementary Materials — Supplementary Figure 1: Fb-Eb calculated with the optB88-vdW functional. Supplementary Figure 2: distribution of energy error (Fbd0/η − Eb). Supplementary Figure 3: distribution of Fbd0/Eb. Supplementary Figure 4: distribution of the calculated d0 and d1. Supplementary Figure 5: diagram of the geometry for the integration model. Supplementary Figure 6: visualization of Supplementary equation (13). Supplementary Figure 7: visualization of I1 − I2 with t = 15 nm. Supplementary Figure 8: XRD characterization of the three different 2D materials. Supplementary Figure 9: SEM characterization (with BSE) of the three fabricated probes. Supplementary Figure 10: high-resolution SEM characterization (with SE) of the fabricated probes. Supplementary Figure 11: comparison of the coated 2D material flakes before and after measurements. Supplementary Table 1: the binding energy and binding force calculated with the vdW-DF2 functional. Supplementary Table 2: the binding energy and binding force calculated with the optB88-vdW functional. [file 9765121.f1.zip › SI-214-F.docx]

Supplementary Information

**High-throughput calculation of interlayer van der Waals forces validated with experimental measurements**

Kewei Tang ^1^, Weihong Qi ^1 *^, Yaru Wei^1^, Guoliang Ru^1^, Weimin Liu^1,2 *^

^1^ State Key Laboratory of Solidification Processing, Center of Advanced Lubrication and Seal Materials, Northwestern Polytechnical University, Xi’an 710072, China

^2^ State Key Laboratory of Solid Lubrication, Lanzhou Institute of Chemical Physics, Chinese Academy of Sciences, Lanzhou 730000, China

*Corresponding Authors: qiwh216@nwpu.edu.cn (W. H. Qi); wmliu@licp.cas.cn (W. M. Liu)

1. **High-throughput Calculation of** $\boldsymbol{F}_{\boldsymbol{b}}$ **and**$\boldsymbol{E}_{\boldsymbol{b}}$
   1. *The results produced by the optB88-vdW functional*

**Supplementary Figure 1** shows the calculated $E_{b}$ and $F_{b}$ for 219 different 2D materials with the optB88-vdW functional. The plot shows a similar trend as **Figure 1c** (vdW-DF2). It is clear that both $E_{b}$ and $F_{b}$ calculated with the optB88-vdW functional are larger than those calculated with the vdW-DF2 functional in general. The number of data points is smaller than that of the vdW-DF2 calculations due to poor convergence and divergence in the case of optB88-vdW calculations.


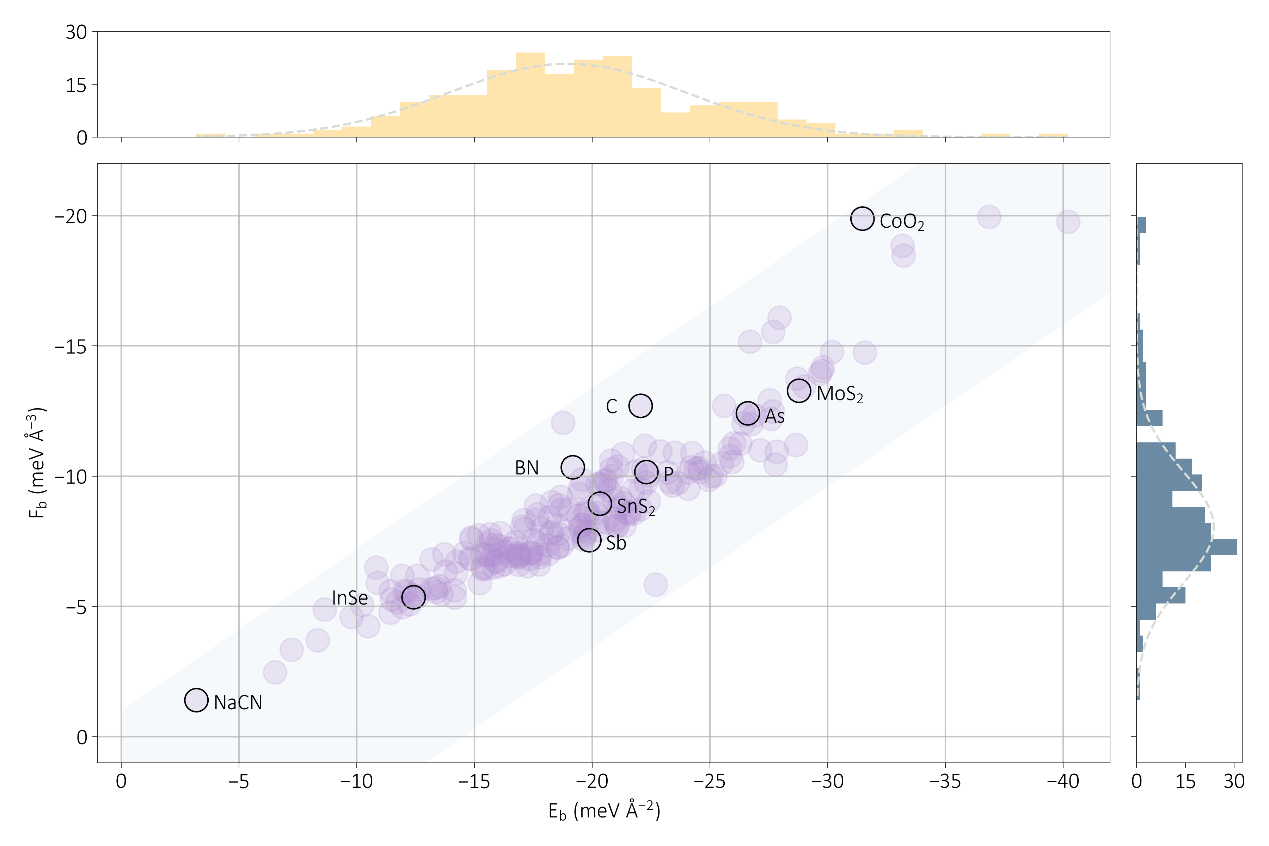


**Supplementary Figure 1.** $\boldsymbol{F}_{\boldsymbol{b}}$**-**$\boldsymbol{E}_{\boldsymbol{b}}$ **calculated with the optB88-vdW functional.**

- 1. *The loose relationship between* $F_{b}$*,* $E_{b}$ *and*$d_{0}$

Assume that the interlayer binding energy profile follows the traditional n-6 potential^1^, which can be viewed as a more flexible form of the L-J potential. The interlayer binding energy $E(d)$ as a function of interlayer distance $d$ and the interlayer binding force $F\left( d \right)$ as the first derivative of $E(d)$ are shown as follows:

$$\begin{aligned} E\left( d \right)=\epsilon_{0}\left[ {\frac{6}{n-6}\left( \frac{\sigma}{d} \right)}^{n}-\frac{n}{n-6}\left( \frac{\sigma}{d} \right)^{6} \right], d>0\#\left( 1 \right) \end{aligned}$$

$$\begin{aligned} E^{'}\left( d \right)=\frac{\epsilon_{0}}{d}\left[ -\frac{6n}{n-6}\left( \frac{\sigma}{d} \right)^{n}+\frac{6n}{n-6}\left( \frac{\sigma}{d} \right)^{6} \right], d>0\#\left( 2 \right) \end{aligned}$$

$$\begin{aligned} F\left( d \right)=-E^{'}\left( d \right),\#\left( 3 \right) \end{aligned}$$

where $\epsilon_{0}$ and $\sigma$ are undetermined constants. By solving $E'(d_{0})=0$, the equilibrium interlayer distance $d_{0}$ can be obtained:

$$\begin{aligned} d_{0}=\sigma.\#\left( 4 \right) \end{aligned}$$

Then, the equilibrium binding energy $E_{b}$ can be yielded as

$$\begin{aligned} E_{b}=E\left( d_{0} \right)=-\epsilon_{0}.\#\left( 5 \right) \end{aligned}$$

Similarly, by solving $E''(d_{1})=0$, the critical interlayer binding force $F_{b}$ and its corresponding critical interlayer distance $d_{1}$ can be yielded as

$$\begin{aligned} d_{1}=\left( \frac{n}{6} \right)^{\frac{1}{n-6}}\sigma,\#\left( 6 \right) \end{aligned}$$

$$\begin{aligned} F_{b}=F\left( d_{1} \right)=\frac{6n}{n-6}\cdot\left[ -\left( \frac{6}{n} \right)^{\frac{n+1}{n-6}}+\left( \frac{6}{n} \right)^{\frac{7}{n-6}} \right]\cdot\frac{\epsilon_{0}}{\sigma}.\#\left( 7 \right) \end{aligned}$$

**Equation (1)** in the main text is then yielded by combining **Supplementary** **equation (4)**, **Supplementary equation (5),** and **Supplementary equation (7)**. It shows a clear linear relationship between $F_{b}$ and $\frac{E_{b}}{d_{0}}$.

**Supplementary Figure 2** shows the energy error (x-axis) of the fittings in **Figure 2**, which represents the error for predicting $E_{b}$ from $F_{b}$. The error distribution shown is very similar to that of the force error (y-axis) in **Figure 2**. **Supplementary Figure 3** shows the distribution of $\frac{\boldsymbol{F}_{\boldsymbol{b}}\boldsymbol{d}_{\boldsymbol{0}}}{\boldsymbol{E}_{\boldsymbol{b}}}$**.** These results indicate that it is also plausible to roughly predict $E_{b}$ from $F_{b}$ with the proposed relationship.


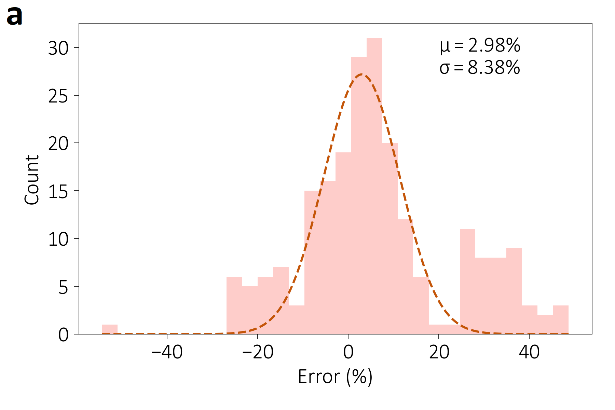

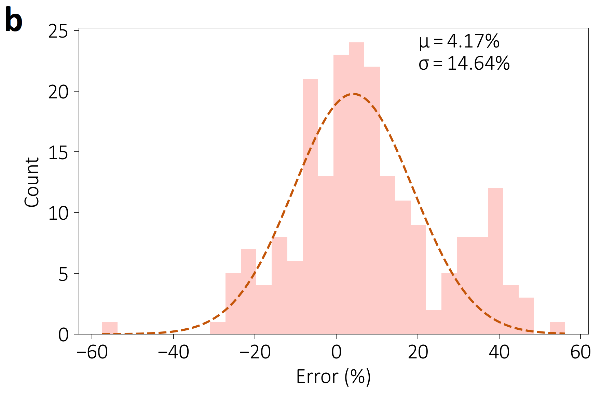


**Supplementary Figure 2. Distribution of energy error (**$\frac{\boldsymbol{F}_{\boldsymbol{b}}\boldsymbol{d}_{\boldsymbol{0}}}{\boldsymbol{\eta}}\boldsymbol{-}\boldsymbol{E}_{\boldsymbol{b}}$**).** **a** vdW-DF2 results. **b** optB88-vdW results (dashed lines are Gaussian fitting). (μ and σ represent the expected value and standard deviation, respectively).


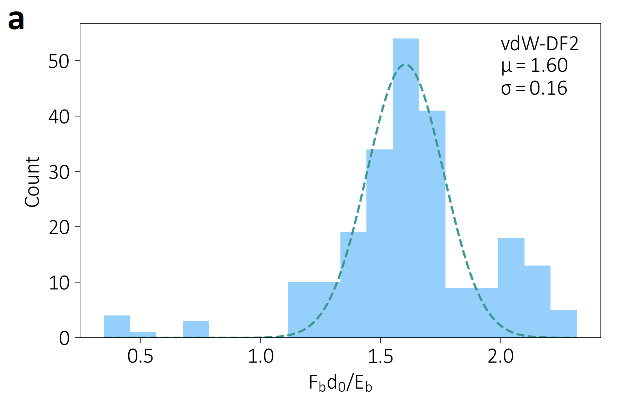

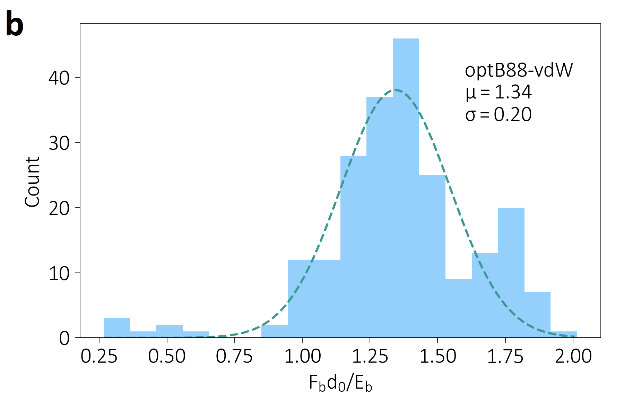


**Supplementary Figure 3. Distribution of** $\boldsymbol{F}_{\boldsymbol{b}}\boldsymbol{d}_{\boldsymbol{0}}\boldsymbol{/}\boldsymbol{E}_{\boldsymbol{b}}$**. a** vdW-DF2 results. **b** optB88-vdW results (dashed lines are Gaussian fitting). (μ and σ represent the expected value and standard deviation, respectively).

- 1. *Calculated* $d_{0}$ *and*$d_{1}$


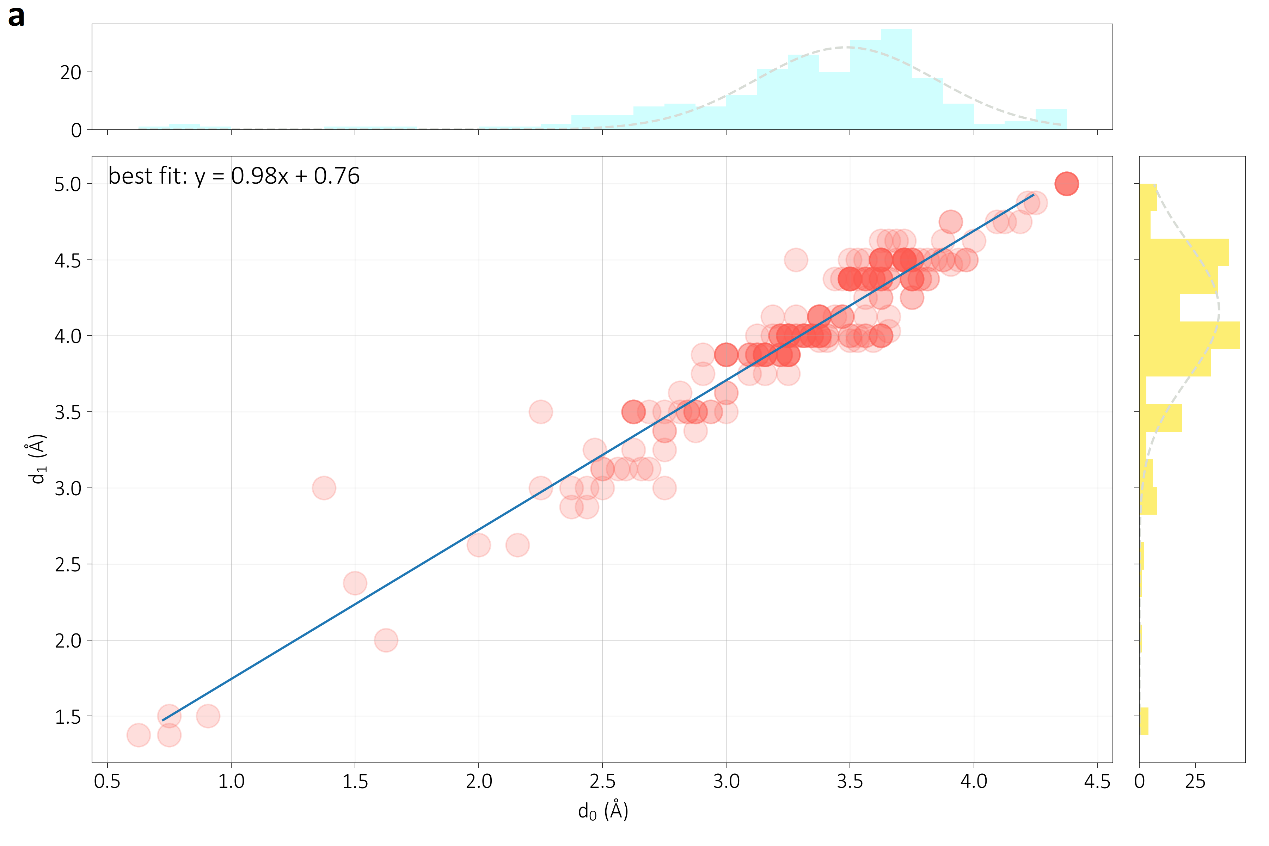


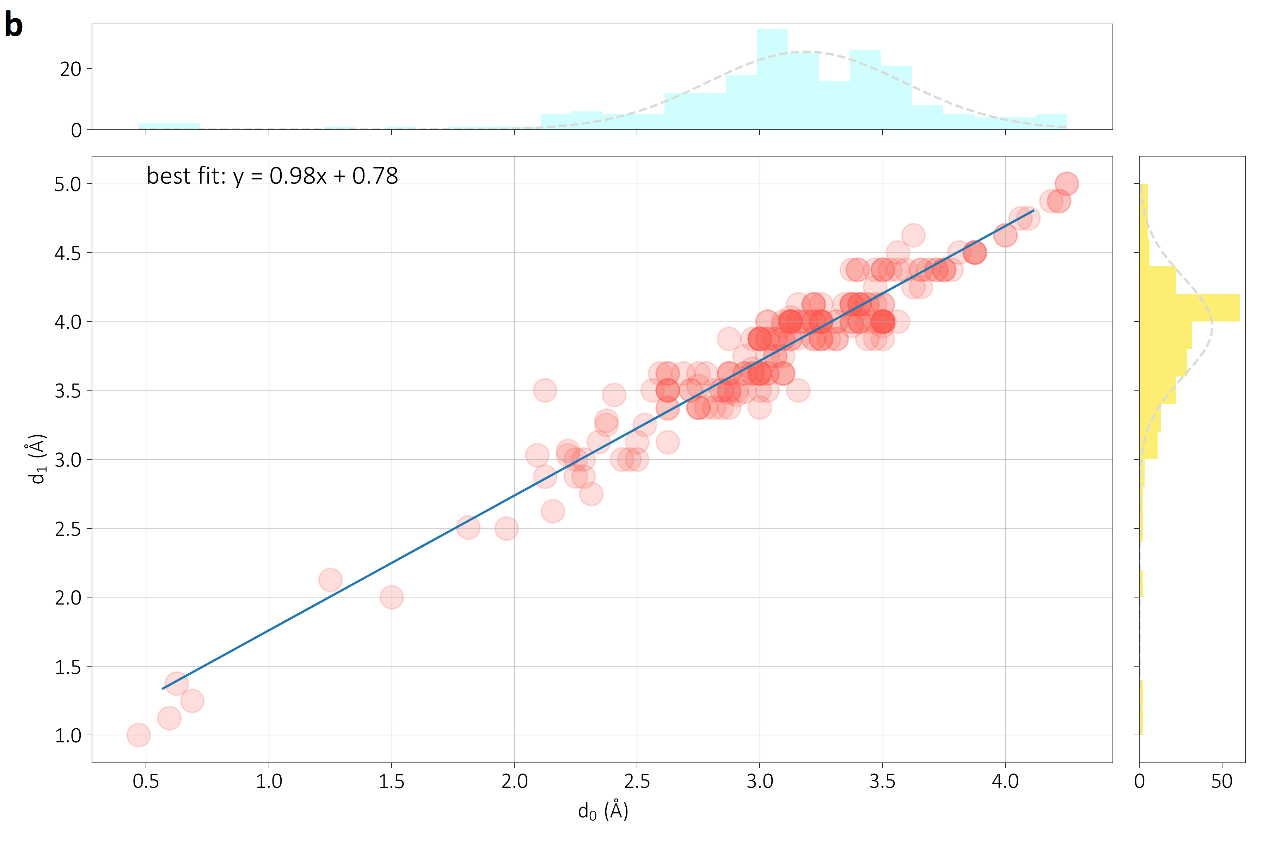


**Supplementary Figure 4. Distribution of the calculated** $\boldsymbol{d}_{\boldsymbol{0}}$ **and** $\boldsymbol{d}_{\boldsymbol{1}}$. **a** The vdW-DF2 results. **b** The optB88-vdW results. The two sets of data are fitted with a line that is rendered in blue and labeled at the top left corner.

The calculated $d_{0}$ and $d_{1}$ are shown in **Supplementary Figure 4**. The equilibrium interlayer distance $d_{0}$ is distributed mainly in the range of 3.0 to 4.0 from the vdW-DF2 results and 2.5 to 3.5 from the optB88-vdW results, while the critical interlayer distance $d_{1}$ is distributed mainly in the range of 3.5 to 4.5 from the results of both functionals. The outstanding data points located at the bottom left corner are the 2D hydroxides, as discussed in the main text. From **Supplementary equation (4)** and **Supplementary equation (6)**, the two distances $d_{0}$ and $d_{1}$ should have a proportional relationship ($d_{1}=1.11d_{0}$). However, through careful examination of the data, it was found that the two quantities fit better with a line, not through the origin. The data from both of the functionals show a best fit slope of ~0.98, with an intercept of ~0.77.

1. **Modeling the vdW interaction between the 2D material probe and substrate**
   1. *General model for the coated spherical probe*


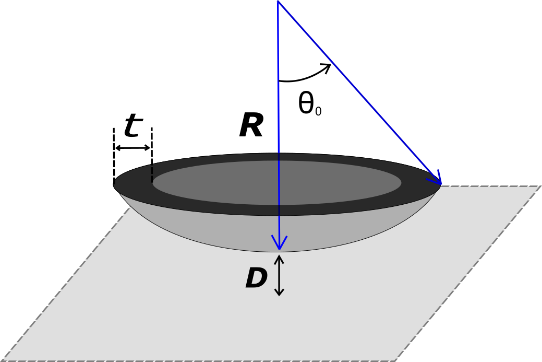


**Supplementary Figure 5. Diagram of the geometry for the integration model.** $R$ is the curvature radius of the coated material, $t$ is the shell thickness of the coated material, $\theta_{0}$ controls the size of the coated material and $D$ is the distance between the probe and the underlying substrate (the substrate is assumed to be infinite in plane and in depth).

The materials coated on the spherical probe on the substrate of the same kind of material are modeled as spherical shells, as shown in **Supplementary Figure 5**. Since the dispersive term is the dominant term near the distance we are interested in (*d*_1_), only the dispersive term is considered here. The overall vdW energy is represented by integrating the vdW interaction using the L-J potential:

$$\begin{aligned} E_{vdW}\left( D \right)=\boldsymbol{I}_{0}=\int_{0}^{\theta_{0}} \int_{0}^{2\pi} \int_{R-t}^{R} \frac{-C\rho^{2}\pi}{6}\cdot\frac{r^{2}\sin\theta}{\left( D+R-r\cos\theta\right)^{3}}\cdot d\theta d\phi dr \#\left( 8 \right) \end{aligned}$$

$$\begin{aligned} \boldsymbol{I}_{0}= -\frac{C\rho^{2}\pi^{2}}{6}\int_{R-t}^{R} \left( \frac{r}{\left( D+R-r \right)^{2}}-\frac{r}{\left( D+R-r\cos\theta_{0} \right)^{2}} \right)\cdot dr=-\frac{C\rho^{2}\pi^{2}}{6}\left( \boldsymbol{I}_{1}-\boldsymbol{I}_{2} \right) \#\left( 9 \right) \end{aligned}$$

$$\begin{aligned} \boldsymbol{I}_{2}=\left\{ \begin{aligned} \frac{1}{\cos^{2} \theta_{0}}\left\{ \begin{aligned} \ln\frac{D+R-R\cos\theta_{0}}{D+R-\left( R-t \right)\cos\theta_{0}}- \\ \left( D+R \right)\left( \frac{1}{D+R-\left( R-t \right)\cos\theta_{0}}-\frac{1}{D+R-R\cos\theta_{0}} \right) \end{aligned} \right\}\left( \cos\theta_{0}\neq0 \right) \\ \frac{R^{2}-\left( R-t \right)^{2}}{2\left( D+R \right)^{2}} \left( \cos\theta_{0}=0 \right) \end{aligned} \right.\#\left( 10 \right) \end{aligned}$$

$$\begin{aligned} \boldsymbol{I}_{1}=\boldsymbol{I}_{2}\left. \right|_{\cos\theta_{0}=1}=\ln\frac{D}{D+t}-\left( D+R \right)\left( \frac{1}{D+t}-\frac{1}{D} \right) \#\left( 11 \right) \end{aligned}$$

where $\rho$ is the material density and $C$ is a constant. By setting $t=R$, $\theta_{0}=\pi$, the integration represents the vdW energy between a spherical tip (ball) and an infinite atom sheet. Considering $R\gg D$ (the $D$ we are interested in is in the magnitude of angstrom), the integration is approximated as

$$\begin{aligned} E_{vdW}\left( D \right)=\frac{C\rho^{2}\pi^{2}}{6}\left[ \ln\left( \frac{D}{D+2R} \right)+\left( D+R \right)\left( \frac{1}{D}-\frac{1}{D+2R} \right) \right]\cong\frac{A_{H}R}{6D} \#\left( 12 \right) \end{aligned}$$

where $A_{H}$ is the Hamaker constant ($A_{H}=C\rho^{2}\pi^{2}$). The above expression is identical to that given by the Derjaguin approximation^2^.

- 1. *Shell thickness independence*

By setting $\theta_{0}$ = π, the vdW energy between a complete spherical shell with thickness $t$ and an infinite atom sheet can be derived:

$$\begin{aligned} E_{vdW}\left( D \right)=\frac{C\rho^{2}\pi^{2}}{6}\left[ \ln\left( \frac{D}{D+t}\cdot\frac{D+2R-t}{D+2R} \right)+\left( D+R \right)\left( \frac{1}{D}-\frac{1}{D+t}+\frac{1}{D+2R-t}-\frac{1}{D+2R} \right) \right] \#\left( 13 \right) \end{aligned}$$

Setting $R$ = 4.5 μm, which is our case, the expression is visualized,


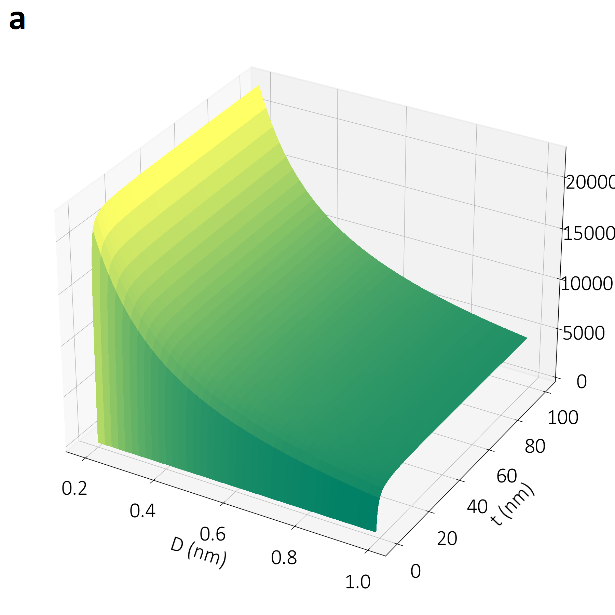

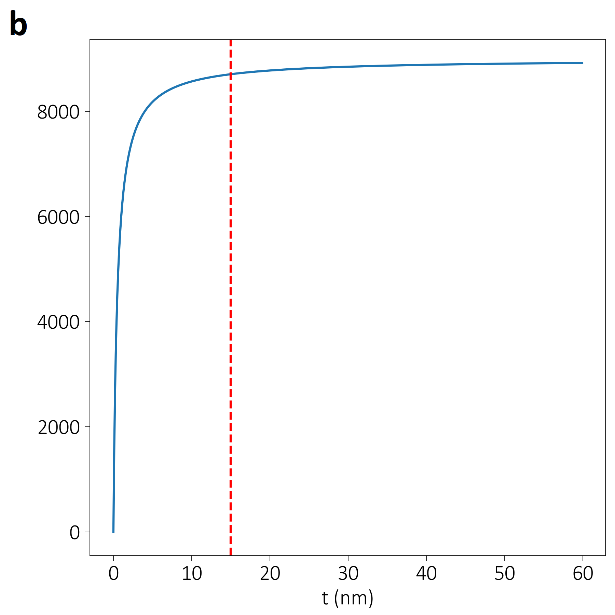


**Supplementary Figure 6. Visualization of Supplementary equation (13). a** The vdW energy (dimensionless) varying with distance and probe shell thickness. **b** The vdW energy (dimensionless) varying with shell thickness at $D$ = 5 Å.

From the above figure, it can be seen that within the distance region (0.2~1.0 nm) that we are most interested in, the majority of the contribution comes from the outermost part of the probe. More specifically, $E_{vdW}(D)$ ceases to change with respect to the probe shell thickness ($t$) after it reaches a sufficiently large value. This indicates that given that the 2D materials capped on the probe are thick enough, the variation of its thickness will not affect the energy. In fact, by assuming $R\gg t$ and $t\gg D$, **Supplementary equation (13)** can be simplified as follows:

$$\begin{aligned} E\left( D \right)\cong\frac{A_{H}}{6}\cdot\left( \frac{R}{D}-\ln\frac{2R+t\left( 1+2\frac{R}{D} \right)}{2R-t} \right) \#\left( 14 \right) \end{aligned}$$

While the first term represents a full-ball contribution, the second term serves as a “thickness dependent correction”. However, since the expression in the logarithmic term is linear to $\frac{R}{D}$, its contribution is negligible to the first term given that $R\gg D$, indicating that thickness will not affect binding energy. In fact, by setting $D$ = 5 Å, which is close to $d_{1}$, **Supplementary Figure 6b** shows that when the thickness is larger than ~15 nm, the energy varies almost linearly with the shell thickness, which indicates that when the shell thickness is large enough, the measured forces will not be affected by it.

In conclusion, under the conditions of $R\gg t$ and $t\gg D$, the vdW energy and force measured by a 2D-material-coated spherical probe are identical to those measured by a solid spherical probe made with all 2D materials. In short, the thickness of the coating material will not affect the measured force if it is significantly larger than $d_{0}$ or $d_{1}$.

- 1. *Shell size independence*

It is clear that the contribution of the integration comes mainly from the central part of the surface of the probe, which is closest to the underlying atom sheet. However, knowing the exact contribution distribution can help us understand how the shell size will affect the measurement results. Since it has been demonstrated that a $t$ larger than approximately 15 nm makes no difference in the integration, we choose $t$ = 15 nm and plot $\boldsymbol{I}_{1}-\boldsymbol{I}_{2}$ in **Supplementary equation (9)** as a function of $D$ and $\theta_{0}$ (this angle determines the size of the coated material):


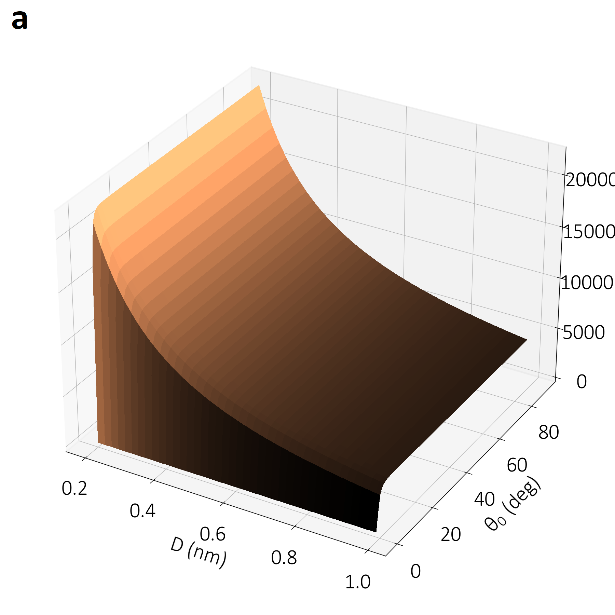

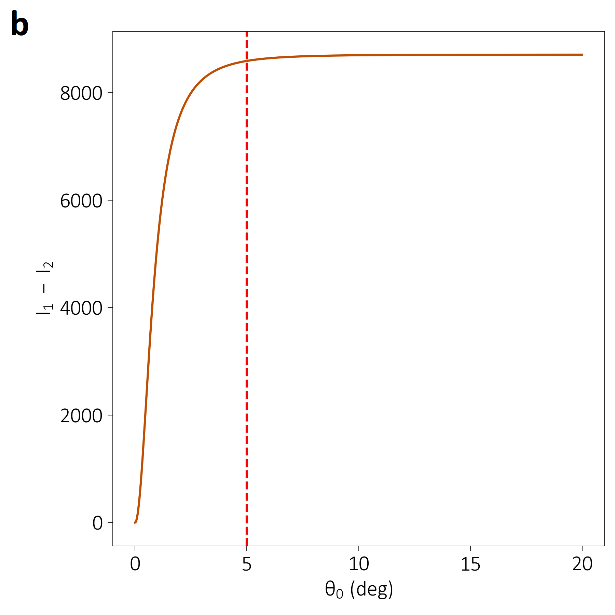


**Supplementary Figure 7. Visualization of** $\boldsymbol{I}_{\boldsymbol{1}}\boldsymbol{-}\boldsymbol{I}_{\boldsymbol{2}}$ **with** $\boldsymbol{t}$ **= 15 nm. a** $I_{1}-I_{2}$ varying with distance $D$ and angle $\theta_{0}$. **b** $I_{1}-I_{2}$ varying with angle $\theta_{0}$ when setting $D$ = 5 Å.

**Supplementary Figure 7a** shows that within the distance range that we are interested in, the majority of the integration contribution comes from the central part of the coated material within the region of approximately $\theta_{0}$ < 5°. **Supplementary Figure 7b** shows a special case for $D$ = 5 Å. Obviously, when $\theta_{0}$ reaches ~5 degrees, the integration has reached very close to its maximum and varies little with increasing $\theta_{0}$. This indicates that both the energy and force are not affected by $\theta_{0}$ when this angle is large enough. The shell size corresponding to $\theta_{0}$ = 5° is approximately 0.60 μm^2^, calculated with $R$ = 4.5 μm. This means that the exact size and shape of the coated material is irrelevant to the results if it covers a sufficient area (~0.60 μm^2^) around the tip.

- 1. *Designing the measurement*

The experiments in this work are carried out by using AFM with 2D-material-coated probes to perform normal-force measurements on 2D material surfaces. Since it is extremely difficult to precisely control the size, shape, and thickness of 2D materials coated on a probe, we chose to use spherical probes. To make the coating process plausible, the spherical probe must be large enough. Hence, the spherical probes are actually assembled with tipless cantilevers and silica balls with a uniform radius of ~4.5 μm. By using a spherical probe, the size and shape of the coated 2D materials are expected to affect the measurement results less. By modeling the interaction between the coated 2D materials and the 2D material substrate under the probe, the influence of the size, shape and thickness of the coating material is closely investigated. The analysis in the previous subsections concludes that given that the thickness of the coated 2D materials is large enough (>15 nm), the measured force is irrelevant to it. The shape and size of the coated material will not affect the results if the flake covers enough area around the tip (~0.6 μm^2^). Regarding flake thickness, 2D materials can be prepared thick enough very easily. Regarding the flake shape and size, the influence can be eliminated by aligning the flake precisely to the center of the silica ball. However, there is still roughness on both sides of the contacting interface to be considered. This concern is public and can hardly be prevented. Although there are models and methods proposed to account for the effect of roughness in force measurements^3,4^, these methods will not be accurate. For simplicity and effectiveness, in this work, the influence of roughness is moderated by performing measurements at different large areas and collecting massive results for Gaussian fitting to produce expected values.

To address the influence of curvature differences, the forces can be corrected proportionally to the differences of $R$ (since $F\cong\frac{A_{H}R}{6D^{2}}$, as we have discussed),

$$\begin{aligned} F_{corrected}=\frac{R}{\tilde{R}}\cdot F_{raw}\#\left( 15 \right) \end{aligned}$$

where $\tilde{R}$ is the average radius of all the probes. The radii of the three silica balls can be measured in SEM graphs.

To obtain a rough estimation of the per-unit-area force, the effective contact area $\bar{A}$ is estimated by summing the effective area determined by the vdW energy layer by layer (the repelling term is not included since the vdW term is dominant near $d_{1}$),

$$\begin{aligned} \bar{A}=\sum_{i=0}^{N} \pi\left( R^{2}-\left( R-i\Delta d \right)^{2} \right)\cdot\left( \frac{d_{1}}{d_{1}+i\Delta d} \right)^{7}\#\left( 16 \right) \end{aligned}$$

where $\Delta d$ is the average interlayer distance (including the layer thickness here). We use a discrete expression because the continuous form will likely overestimate the result at small interlayer distances. Setting $\Delta d$ = 3.5 Å, $d_{1}$ = 3.5 Å and $R$ = 4.5 μm, summing the expression to a $N$ corresponding to $\theta_{0}$ = 5°, we obtain $\bar{A}$ = 89.00 nm^2^. Using this value, the per-unit-area forces can be obtained from the directly measured results.

1. **Characterization results**


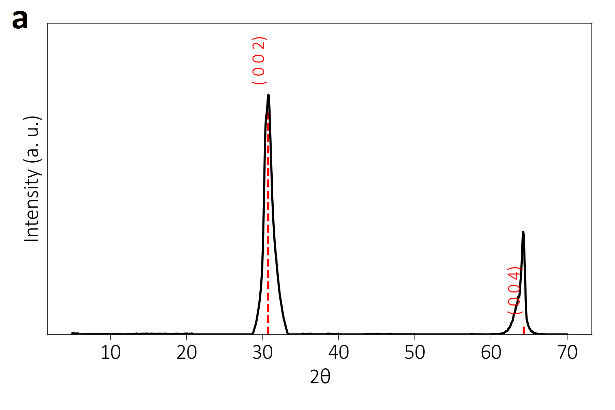

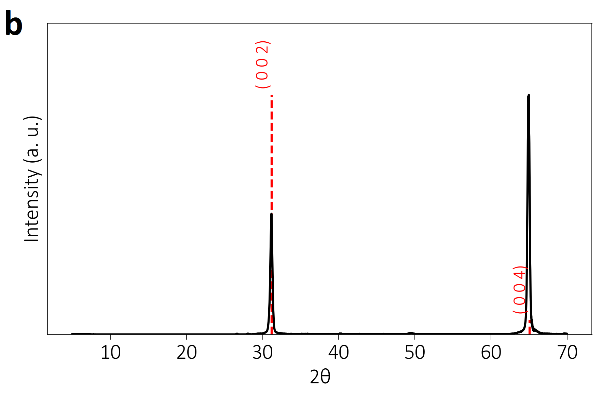


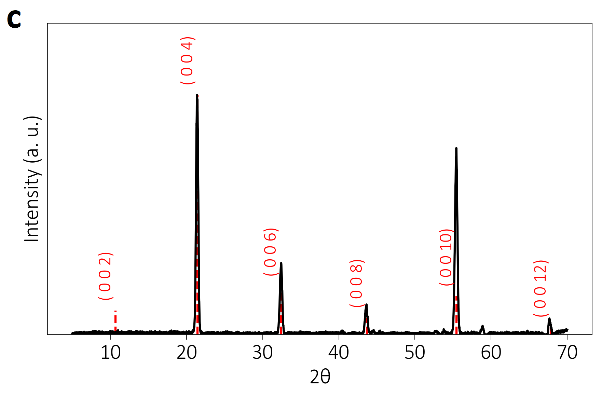


**Supplementary Figure 8. XRD characterization of the three different 2D materials. a** C. **b** BN. **c.** α-In_2_Se_3_. Red lines represent the peaks in the standard PDF.


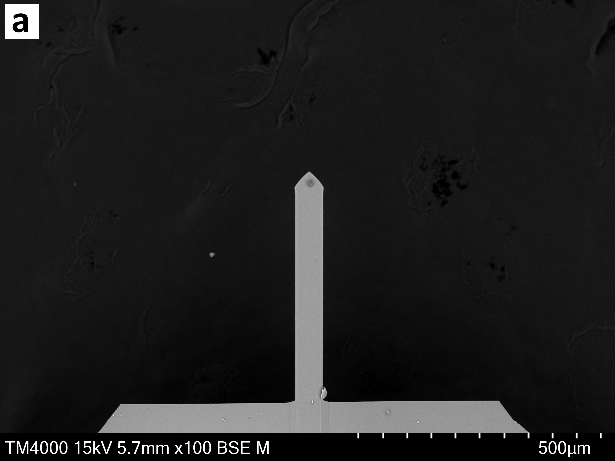

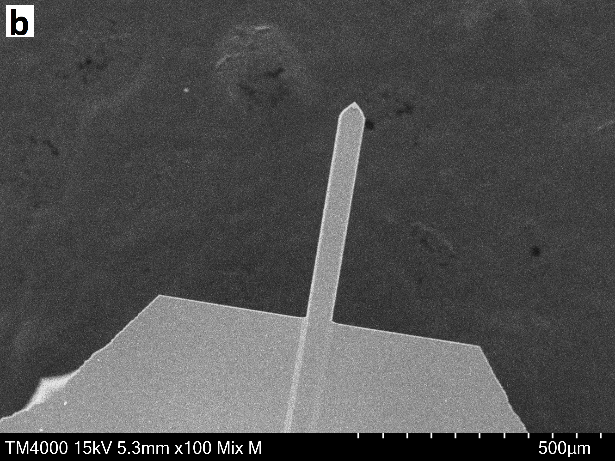

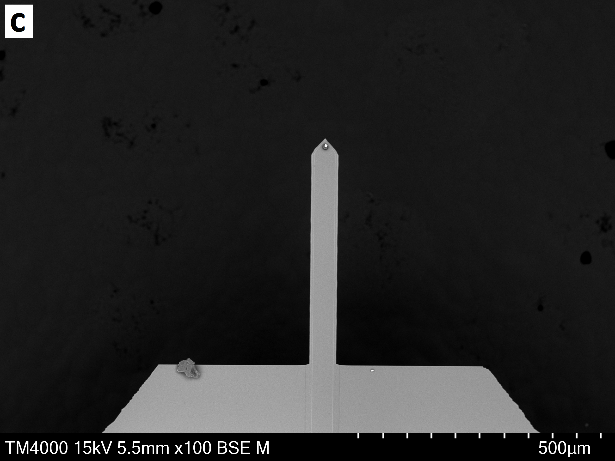


**Supplementary Figure 9. SEM characterization (with BSE) of the three fabricated probes. a** C-coated probe. **b** BN-coated probe. **c** In_2_Se_3_-coated probe.


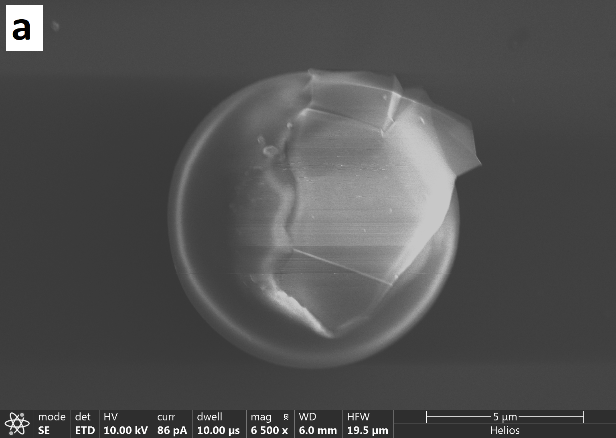

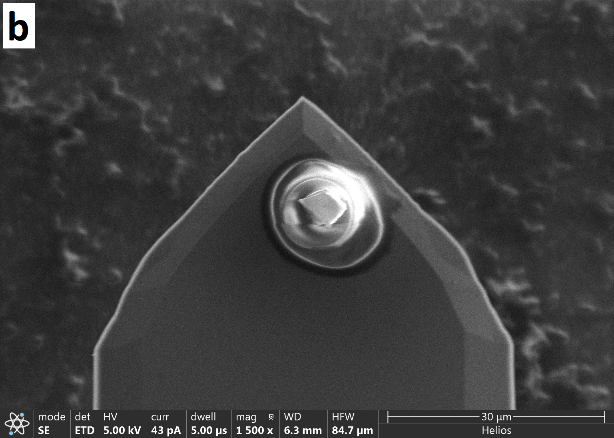


**Supplementary Figure 10. High-resolution SEM characterization (with SE) of the fabricated probes. a** C-coated probe. **b** In_2_Se_3_-coated probe.

The X-ray diffraction (XRD) results are compared with the standard powder diffraction file (PDF), as shown in **Supplementary Figure 8**. The results indicate that the 2D materials used in this work are highly oriented. **Supplementary Figure 9** shows the fabricated cantilevers. The silica balls are well aligned at the top of the cantilevers, and the surfaces of the cantilevers are very clean (not contaminated due to our precise assembly process). **Supplementary Figure 10** shows the high-resolution SEM graphs of the fabricated probes complementary to **Figure 3b**. The 2D material flakes are aligned very well to the center of the silica balls, which cover enough area to ensure that the small misalignment and shape differences of the flakes do not affect the measurement results.


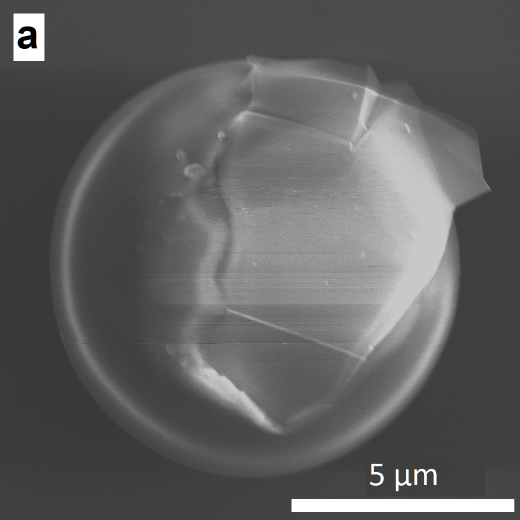

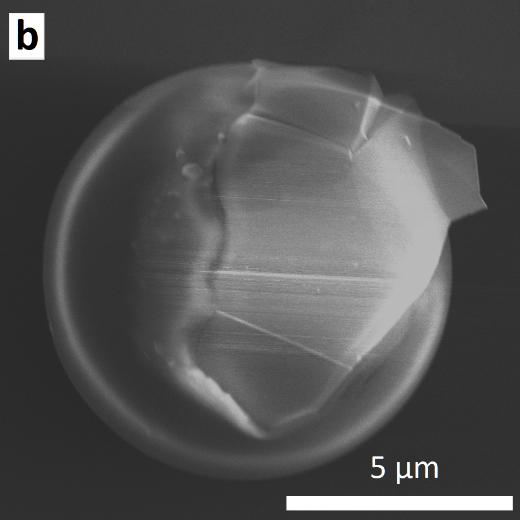


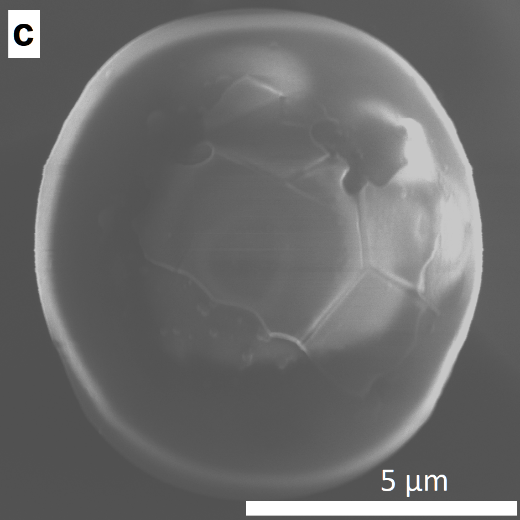

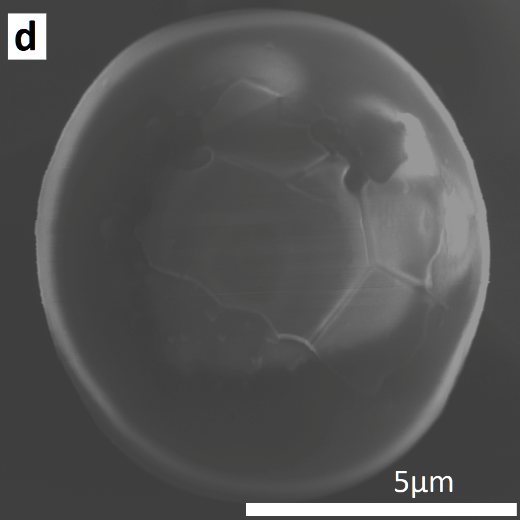


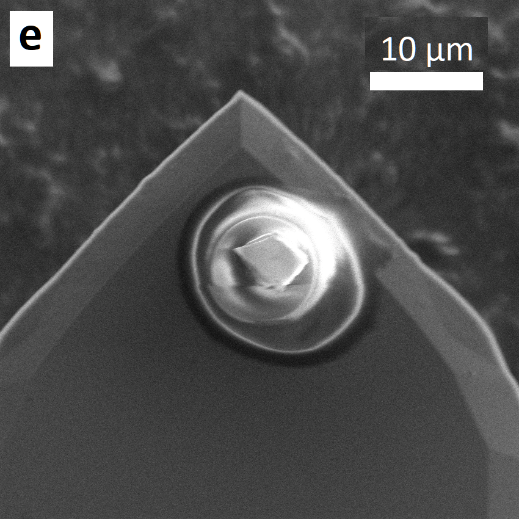

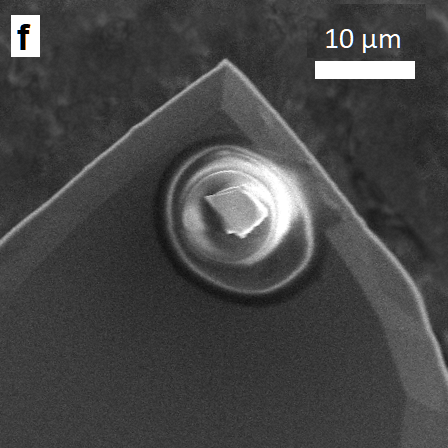


**Supplementary Figure 11. Comparison of the coated 2D material flakes before and after measurements. a** C before measurements. **b** C after measurements. **c** BN before measurements. **d** BN after measurements. **e** In_2_Se_3_ before measurements. **f** In_2_Se_3_ after measurements.

**Figure 11** shows the SEM images for the C (first row), BN (second row), and In_2_Se_3_ (last row) flakes coated on silica balls before (left column) and after (right column) the AFM measurements. This clearly indicates that the 2D material flakes are tightly integrated with the silica balls they did not fall off or undergo any plastic deformation during the tests. It also shows that no extra flakes were added to the probes during the tests.

1. **Discussion on the effect of surface contamination**

Surface contamination is inevitable for both the substrates and probes. For substrates, the main concern is the residual glue induced by tapes. To examine its effects, we performed measurements on exfoliated graphite and nonexfoliated HOPG using a noncoated silica probe. The result is 36.12± 5.22 nN for exfoliated graphite and 35.61± 5.84 nN for nonexfoliated HOPG. The difference is very small, which suggests that the effect of residual glue is negligible. For probes, residual polymer should be considered. PDMS is somewhat sticky and should have a notably larger adhesion force. The adhesion force between a piece of PDMS film and a standard probe is approximately 100-200 nN, as reported by an AFM study^5^. Considering the significantly larger contact area using our homemade probe, the adhesion force should be even much larger than this. However, in our graphite/noncoated silica probe measurement, the result is only 35.61 nN. It would not be this small if there were a considerable amount of PDMS on the surface. Since these contaminations are considered trivial, they can be safely viewed as a systematic error in this study.

**References**

1 Maitland, G. C. & Smith, E. B. A simplified representation of intermolecular potential energy. *Chemical Physics Letters* **22**, 443-446 (1973).

2 Butt, H.-J., Cappella, B. & Kappl, M. Force measurements with the atomic force microscope: Technique, interpretation and applications. *Surface Science Reports* **59**, 1-152 (2005).

3 Rabinovich, Y. I., Adler, J. J., Ata, A., Singh, R. K. & Moudgil, B. M. Adhesion between Nanoscale Rough Surfaces: I. Role of Asperity Geometry. *J. Colloid Interf. Sci.* **232**, 10-16 (2000).

4 Jacobs, T. D. B. *et al.* The Effect of Atomic-Scale Roughness on the Adhesion of Nanoscale Asperities: A Combined Simulation and Experimental Investigation. *Tribol. Lett.* **50**, 81-93 (2013).

5 Meincken, M., Berhane, T. A. & Mallon, P. E. Tracking the hydrophobicity recovery of PDMS compounds using the adhesive force determined by AFM force distance measurements. *Polymer* **46**, 203-208 (2005).
